# Modified Dice Coefficients for Evaluation of Tumor Segmentation from PET Images: A Proof-of-Concept Study

**DOI:** 10.1007/s10278-025-01535-1

**Published:** 2025-05-08

**Authors:** Oona Rainio, Riku Klén

**Affiliations:** Turku PET Centre, University of Turku and Turku University Hospital, Turku, Finland

**Keywords:** Convolutional neural network, Dice coefficient, Medical imaging, Positron emission tomography, Tumor segmentation

## Abstract

The Sørensen-Dice similarity coefficient (DSC) is the most common evaluation metric used for image segmentation but it is not always ideal. Namely, the DSC values only depend on the number of misplaced elements instead of their location with respect to the correct segments. Because of this, the DSC is ill-suited for such tasks where the correct location of the borders of an object is difficult to define in an objective way, as is the case in tumor segmentation in positron emission tomography (PET) images. To avoid this issue, we introduce two different modifications of the DSC, one with weights and one with an additional loss term, which also evaluate the distance between the real and the predicted segments. We computed the values of DSC and our new coefficient from 191 predicted tumor segmentation masks created by using PET images of 89 head and neck squamous cell carcinoma patients. We compared the values of all three coefficients with the scores given to these masks by human evaluators. According to our results, the weighted modification of DSC had a higher correlation with the scores given by the human evaluators than the original DSC, and it also produced significantly less variation within the two highest score classes (*p*-value$\le$0.018). The new weighted coefficient introduced here has much potential in the evaluation of segmentation results from medical imaging.

## Introduction

Image segmentation is one of the standard tasks performed with image-based deep learning techniques, such as a convolutional neural network (CNN), to locate different objects and regions and their boundaries in images. It is commonly utilized, for instance, to denote anatomical structures in medical images [1–3], to point out possible intruders or other threats in video surveillance [4], or to give information about surrounding traffic to self-driving cars [5]. To evaluate the results of image segmentation, we need to estimate the likeness between the real segment *X* annotated by a human and the predicted segment *Y* given by some machine-learning technique. The most common method for this purpose is Sørensen-Dice similarity coefficient (DSC) [6, 7], also known as the Dice score, defined as

1.1 $$\begin{aligned} \textrm{DSC}=\frac{2|X\cap Y|}{|X|+|Y|}\in [0,1], \end{aligned}$$

where |*X*| denotes the number of pixels or voxels in the set *X* and $\cap$ is the intersection of two sets.

**Fig. 1:**
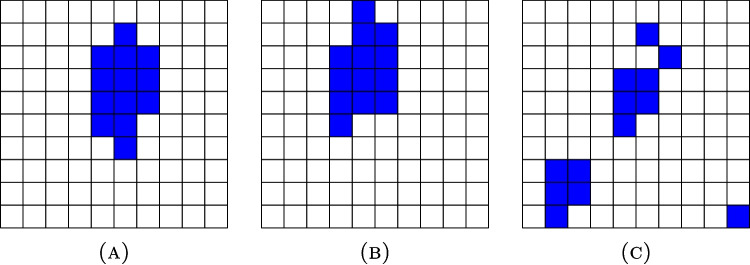
Three segmentation masks, in which the positive pixels are denoted with blue color. The Dice score between masks **A** and **B** is 0.538, which is the same as the Dice score between masks **A** and **C**. However, visually the mask B clearly resembles the mask **A** more than the mask **C** does

As a measure of the amount of relative overlap, the DSC fits the intuitive idea of a measurement of similarity. Its interpretation is simple: The value 1 means perfect resemblance and 0 means that there is no overlap. However, all the elements outside the overlap of the segments have the same weight. Consequently, only the number of misplaced pixels or voxels matters, not their location. As shown in Fig. 1, predicted segmentation masks produce the same DSC regardless of whether the incorrect points are located near the boundary or completely outside of the real object. This is especially problematic for tumor segmentation because certain medical imaging techniques, such as positron emission tomography (PET), do not produce images with sharp boundaries between different regions due to the partial volume effect. Instead, the predicted outline of the tumor often varies even when annotated by multiple radiologists.

While the use of DSC has some existing alternatives, they either suffer the issue as DSC or do not measure the overlap. The problem might be even more pronounced for surface or boundary-based metrics such as surface Dice score or boundary F1 score: If the ground-truth boundary location cannot be defined in an objective way, these metrics can give very low values on such segmentation results where the tumor is correctly placed according to another radiologist. While we can use Hausdorff distance [8] or other such metrics that estimate the distance between the predicted segments, they cannot be used to replace the DSC. Additionally, while possible, the combination of two metrics is not ideal if we want to measure both the overlap and distance between the segments by a single metric for the sake of statistical testing.

One possible solution would be to modify the definition of the original DSC so that a falsely labeled pixel produces the greater error the further away it is from the corrected labeled positive pixels. This can be implemented by using dilatation in weighting of the pixels so that the original pixels of the sets *X* and *Y* have weight 1 and their environments have some smaller positive weight. An alternative approach would be to use an additional loss term in the DSC to define a higher penalty for the false pixels that are too far away from the correct segment.

In this proof-of-concept article, we introduce two modifications of the DSC, one of which is based on weighting the dilation of the original segment and one of which uses a loss term for the pixels too far outside of the correct segments. We compare their values to those of the original DSC computed from the tumor masks predicted by a CNN from PET images of head and neck cancer patients. Our aim is to find a coefficient which could easily be interpreted in a way that corresponds with the visual evaluation of the segmentation results by a human.

## Material and Methods

### Software Requirements

Our experiment was run in Python (version: 3.9.9) [9] by using the packages TensorFlow (version: 2.7.0) [10], Keras (version: 2.7.0) [11], and SciPy (version: 1.7.3) [12]. The PET images were inspected by using Carimas (version: 2.10) [13]. The modified Dice coefficients were computed with SciPy and the basic NumPy functions.

### Data

The data of this study was retrospectively collected from 89 patients diagnosed with head and neck squamous cell carcinoma. After their initial treatment with chemoradiotherapy, they were referred for a PET/magnetic resonance imaging (MRI) treatment response assessment in Turku PET Centre, Turku, Finland, during the years 2014–2022. Their mean age was 62 years with a standard deviation of 12 years, and their male-female sex ratio was 2.1. All the patients were over 18 years of age and gave informed consent to the research use of their data. The data of this study was originally used for the research in [14].

Imaging of the patients was performed with either 3T Philips Ingenuity TF PET/MRI scanner (Philips Health Care) or SIGNA$^{\text {TM}}$ PET/MRI scanner with QuantWorks (GE Healthcare) by using $^{18}$F-fluorodeoxyglucose as tracer substance. All the images depicted the head and neck area of the patients, and the presence of the tumors in the images was confirmed with histopathological sampling or follow-up imaging. We excluded both MRI images and the potential follow-up scans from our data, leaving us with only one PET image from each patient. With the aid of an experienced nuclear medicine physician, a physician created a binar segmentation mask for the PET images by labeling all the voxels depicting cancer as positive and the rest as negative.

**Fig. 2:**
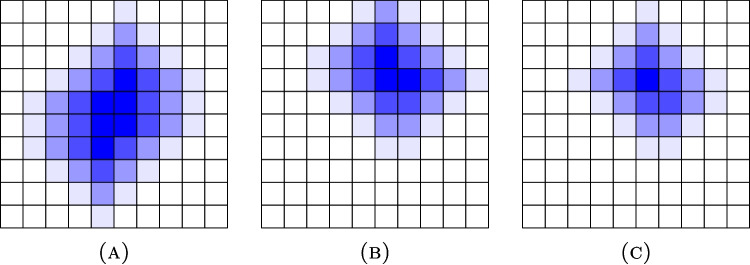
Visualizations of two $10\times 10$ segmentation masks **A** and **B** so that the pixels classified as positive are denoted with the darkest shade of blue and their three environments with lighter shades of blue, and a similar visualization of the element-wise minimum matrix **C**. The Dice score of the masks **A** and **B** is 0.222. If the three environments have weights $\nu _1=0.7$, $\nu _2=0.5$, and $\nu _3=0.3$, then the weighted Dice coefficient of the masks **A** and **B** is 0.600

Since we performed two-dimensional segmentation, the PET images were divided into transaxial slices. Each PET image had 32–66 slices of $512\times 512$ pixels, but these slices were converted into the size of $128\times 128$ as in [15]. The pixel values were scaled onto the interval [0, 1] for each slice separately. Their masks were converted into the same size by using the threshold 0.25 to classify the pixels in the resized masks into positive and negative. All the slices with less than 6 positive pixels according to the new masks were excluded from the data. Our final data had 962 slices from the 89 patients and it was divided patient-wise into a training and a test set so that the set had 191 slices (19.9% of the total data) of 17 patients. After using a CNN to perform the segmentation for the two-dimensional slices, we also combined the predicted and the real masks into 17 pairs of three-dimensional masks to test how the modified Dice coefficients work on three-dimensional data.

### Convolutional Neural Network

To obtain predicted binary masks from the data, we used a CNN whose design follows the U-Net architecture introduced by Ronneberger et al. in 2015 [18]. The CNN first has a contracting path that consists of four sequences containing a convolution layer, a dropout layer, another convolution layer, and a maximum pooling layer. The CNN then has an expanding path of three sequences, each of which contains a transpose convolution layer, concatenation operation for the outputs of the previous layer and one earlier convolution layer of the same dimension from the contraction path, another convolution layer, a dropout layer, and a third convolution layer. The idea behind these two paths is that the U-Net CNN can first see the whole image and then focus on the details needed for the segmentation. All the other layers of the CNN use ReLu activation except the last one, which has a linear function. We used binary cross-entropy as our loss function and the optimizer was Adam with the learning rate of 0.001. The same CNN set-up has been also used in [15–17].

The CNN was trained during 50 epochs with the training data. A 30% of the training data was used for validation to define an early stopping criterion. The convergence was checked visually by plotting the loss function. After the training, both the images of the training data and the test data were predicted. The value 0.40 was used as a threshold to convert the numeric output of the CNN into binary masks because this threshold value resulted in the highest median and mean DSCs (0.759 and 0.671, respectively) from the predictions of the training data.

**Table 1:** The meaning of the scores 0–4, their criteria, and the number of predicted masks which received each score

Score	Meaning	Criteria	Masks
0	Fully	The predicted mask is completely wrong so that it is	25
	wrong	nowhere near the area of the real tumor, or all the pixels	
		are incorrectly predicted as negative	
1	Poor	The predicted mask is located so that it touches the	28
		outline of the real tumor at some places. However	
		most of the areas of the predicted mask and the real	
		mask of the tumor do not overlap. There might be	
		several fully FP or FN components	
2	Decent	The predicted mask is located at the correct place but	45
		its outlines are fully outside or inside the outlines of	
		the mask drawn by a physician. Alternatively, there is	
		at least one fully FP or FN component or other	
		significant error. There can be even two or three FP	
		or FN components but only if they are very small	
		in their size compared to other parts of the masks	
3	Good	The predicted mask only requires slight corrections	40
		The outlines of the predicted positive area and those	
		of the real tumor clearly intersect, and their areas	
		mostly overlap. There are neither fully FP or FN	
		components	
4	Excellent	The predicted mask is perfect or nearly perfect. No	53
		corrections are required. The differences between the	
		predicted mask and the mask drawn by a physician	
		are comparable to the differences between masks	
		drawn by two different physicians	

### Modified Dice Coefficients

A segmentation mask of any two- or three-dimensional image is a binary matrix of the same size, whose each element reveals if the pixel or the voxel at the corresponding location in the image is labeled as positive (value 1) or negative (value 0). There are also multi-class segmentation masks, but we focus on the binary segmentation here. For two segmentation masks, we denote the sets of their elements with values of 1 by *X* and *Y*, respectively. Their DSC is defined as in the formula Eq. 1.1 in the “Introduction’’ section.

Let *n* be an integer. Choose coefficients $0<\nu _n<\nu _{n-1}<...<\nu _1<1$ for indexes $i=1,...,n$. Fix then *n* environments of the sets *X* and *Y* so that $X\subseteq X_1\subseteq ...\subseteq X_n$, and $Y\subseteq Y_1\subseteq ...\subseteq Y_n$. Use the index 0 for the original sets, i.e., $X=X_0$ and $Y=Y_0$. The weighted Dice coefficient (WDC) is now defined as

2.1 $$\begin{aligned} \textrm{WDC}=2\,\frac{|X\cap Y|+\sum ^n_{i=1}\nu _i|X_i\cap Y_i\setminus (X_{i-1}\cap Y_{i-1})|}{|X|+\sum ^n_{i=1}\nu _i|X_i\setminus X_{i-1}|+|Y|+\sum ^n_{i=1}\nu _i|Y_i\setminus Y_{i-1}|}\in [0,1]. \end{aligned}$$

While the above formula might look complicated, the value of WDC can be calculated very efficiently with the following instructions: Create first a matrix of the size of the segmentation mask and assign each element *p* in the matrix a value 1 if $p\in X$, $\nu _i$ if $p\in (X_i\setminus X_{i-1})$ and 0 if $p\notin X_n$. Then create a similar matrix for the set *Y*. Let $s_X$ and $s_Y$ be the sums of all the elements in these two matrices, respectively. Create another matrix of the same size so that it is the element-wise minimum of the other two matrices, and denote the sum of the elements of this matrix by *s* (see Fig. 2). Then, we have

2.2 $$\begin{aligned} \textrm{WDC}=\frac{2s}{s_X+s_Y}. \end{aligned}$$

For *X* and *Y*, choose some environments $X^*$ and $Y^*$ so that $X\subseteq X^*$ and $Y\subseteq Y^*$. Define the loss-based Dice coefficient (LDC) as

2.3 $$\begin{aligned} \textrm{LDC}=\frac{2|X\cap Y|}{|X|+|Y|+|X\setminus Y^*|+|Y\setminus X^*|}\in [0,1]. \end{aligned}$$

This definition is the same as that of the typical DSC except we use the number of elements in the sets *X* and *Y* with a high enough distance from *Y* and *X* to be outside $Y^*$ and $X^*$, respectively, as an additional loss term in the denominator.

In our experiments, we chose $n=3$ and fixed constants $\nu _1=0.7$, $\nu _2=0.5$, $\nu _3=0.3$. There was no preliminary experimentation about the effect of different numerical values for $\nu _i$ and, instead, these specific values were chosen because they divide the interval (0,1) in a simple and symmetric way. The environments of two-dimensional segments *X* and *Y* were chosen as in Fig. 2 so that a pixel *p* belonged to $X_i$ for $i=1,2,3$ if and only $p\in X_{i-1}$ or at least one of the pixels sharing an edge with *p* was in $X_{i-1}$. Similarly, in the three-dimensional case, a voxel *p* belonged to $X_i$ if *p* or a voxel sharing a side with *p* was located in $X_{i-1}$. The binary dilation was coded with the ready function *scipy.morphology.binary_dilation* in our code. We used the sets $X^*=X_3$ and $Y^*=Y_3$ for LDC.

### Scores of the Predicted Masks

The 191 predicted binary tumor masks were given scores from 0 to 4 by using the criteria presented in Table 1 by two different human evaluators. The first evaluator was a postdoctoral researcher with 3 years of research experience on medical image segmentation via deep learning and the second evaluator was an associate professor on 20 years of research experience on image instrumentation and detection technologies. The scores were determined only by visually comparing the predicted binary mask and the real binary mask drawn by a physician without any further information about the DSC values, any other statistics, or the score given by the other evaluator. During this evaluation, we especially considered the number and the size of such components whose every element was either false positive (FP) or false negative (FN). The scores by the first evaluator were considered as ground-truth and the scores by the second evaluator were used to estimate inter-rater agreement and validate the conclusions based on the scores by the first evaluator. Each of the five score classes created according to the first evaluator had 25–53 predicted binary masks. For the three-dimensional masks, we computed the mean value of the scores of the two-dimensional masks forming these three-dimensional masks.

### Statistics

To study the values of the different Dice coefficients within each score class, we used several common statistics. We computed the correlation between the scores and the values of DSC, WDC, and LDC by using Spearman’s rank correlation coefficient as it is better suited for ordinal data than the better-known Pearson’s correlation coefficient. We also performed the typical tests for the significance of correlation. Additionally, we used the F-test of equality of variances to test a hypothesis according to which the amount of variation is the same for two coefficients in a given score class. We rejected the null hypothesis with 5% level of significance. To account for multiple comparison problems related to the repeated use of the *F*-test, we also represented the *p*-values corrected with false discovery rate (FDR) control.

**Table 2:** The minimum, mean, maximum, and standard deviation computed from the values of the original Dice score (DSC), the weighted Dice coefficient (WDC), and the loss-based Dice coefficient (LDC) within each score class of Table 1

Score	Statistic	DSC	WDC	LDC
0	Min	0.000	0.000	0.000
	Mean	0.000	0.002	0.000
	Max	**0**.**000**	0.027	**0**.**000**
	Sd	**0**.**000**	0.006	**0**.**000**
1	Min	0.045	**0**.**142**	0.023
	Mean	0.324	0.410	0.248
	Max	0.857	**0**.**791**	0.811
	Sd	0.214	**0**.**175**	0.197
2	Min	0.091	**0**.**212**	0.054
	Mean	0.545	0.605	0.468
	Max	0.856	0.841	**0**.**824**
	Sd	0.198	**0**.**155**	0.214
3	Min	0.255	**0**.**482**	0.182
	Mean	0.653	0.734	0.620
	Max	0.874	0.891	**0**.**854**
	Sd	0.134	**0**.**091**	0.145
4	Min	0.605	**0**.**747**	0.605
	Mean	0.825	0.874	0.824
	Max	0.980	0.991	0.980
	Sd	0.078	**0**.**053**	0.079

The smallest standard deviation in each class is written in bold. Additionally, the greatest minimum values for scores 1–4 and the smallest maximum values for scores 0–3 are in bold

## Results

**Fig. 3:**
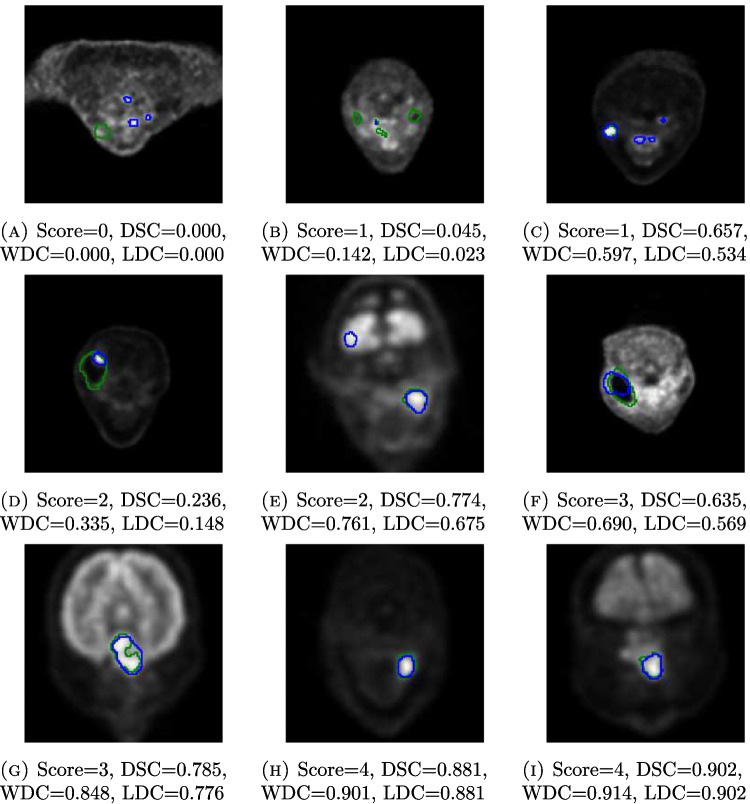
Nine transaxial PET slices where the real tumor mask drawn by a physician is outlined in green and the predicted mask is outlined in blue. The blue outline nearly covers the green one in some cases. The score based on the criteria in Table 1, the original Dice score (DSC), the weighted Dice coefficient (WDC), and the loss-based Dice coefficient (LDC) are denoted under each slice

The minimum, mean, maximum, and standard deviation computed from the values of the DSC, WDC, and LDC within each score class of Table 1 are presented in Table 2. We see that all the values of DSC and LDC for the predicted segmentation masks given the score 0 were 0, but WDC also had some very small ($\le$0.027) positive values within this class. However, with the exception of this first class, WDC always produced the smallest standard deviation within score classes. Its minimum values were typically higher than those of DSC and LDC, and while the maximum of LDC was often smaller, the range of WDC was the smallest within a score class for the scores 1–4. In fact, the difference between the maximum and minimum of WDC within a score class for the scores 1–4 varied from 0.244 to 0.648, while the same differences of DSC and LDC varied between 0.376 and 0.812 and between 0.376 and 0.787, respectively. Spearman’s rank correlation coefficient was 0.832 (*p*-value: 2.88e$-$50) between the DSCs of each predicted segmentation mask and their scores, 0.892 (*p*-value: 2.70e$-$67) between the WDCs and the scores, and 0.867 (*p*-value: 3.62e$-$59) between the LDCs and the scores. Figure 3 shows one example of a transaxial PET slice with both the predicted and the real tumor masks visible for the first score class and two examples for each of the other four score classes, with the corresponding DSC, WDC, and LDC values alongside.

Table 3 contains the original and FDR-corrected *p*-values of the *F*-tests computed from the values of DSC, WDC, and LDC within each score class for the scores 1–4. We could not perform an *F*-test in the score class 0 because all the values of DSC and LDC were 0 and therefore did not follow normally distribution even approximately. From these *p*-values and the standard deviations of Table 2, we can conclude that the variation of the WDC values within a score class is significantly smaller compared to LDC and DSC for scores 3 and 4.

**Table 3:** The original *p*-values/FDR-corrected *p*-values of the F-tests comparing the variances within the score class for the scores 1–4 between the original Dice score (DSC), the weighted Dice coefficient (WDC), and the loss-based Dice coefficient (LDC). The statistically significant *p*-values are in bold

Score	DSC vs WDC	DSC vs LDC	WDC vs LDC
1	0.305/0.523	0.674/0.735	0.543/0.735
2	0.111/0.222	0.613/0.735	**0**.**036**/0.0864
3	**0**.**018**/0.054	0.608/0.735	**0**.**004**/**0**.**024**
4	**0**.**006**/**0**.**024**	0.906/0.906	**0**.**004**/**0**.**024**

**Table 4:** Number of transaxial slices in the 17 three-dimensional segmentation masks, the mean scores of the predicted two-dimensional masks, and the values of the original Dice score (DSC), the weighted Dice coefficient (WDC), and the loss-based Dice coefficient (LDC) computed from the three-dimensional masks

Slices	Score	DSC	WDC	LDC
18	0.8	0.175	0.316	0.105
13	1.2	**0**.**101**	**0**.**192**	**0**.**057**
3	1.3	0.365	0.492	0.235
21	1.5	0.431	0.526	0.336
5	2.0	0.599	**0**.**525**	0.461
6	2.2	0.603	0.600	0.528
9	2.3	0.703	0.675	0.614
13	2.4	0.722	0.794	0.703
15	2.5	**0**.**505**	**0**.**602**	**0**.**424**
18	2.5	**0**.**640**	**0**.**730**	**0**.**605**
13	2.8	0.744	0.826	0.726
7	3.0	**0**.**682**	**0**.**722**	**0**.**641**
16	3.1	0.751	**0**.**798**	0.731
18	3.5	0.779	0.865	0.779
5	3.6	**0**.**710**	**0**.**795**	**0**.**702**
6	3.7	0.819	0.890	0.819
5	3.8	**0**.**789**	**0**.**879**	**0**.**789**

The values of the coefficients that differ from the order of the mean scores are in bold

Table 4 has the values of DSC, WDC, and LDC between the 17 pairs of three-dimensional masks created by combining the predicted two-dimensional segmentation masks and those drawn by a physician for each patient in the test set. By definition of LDC, we know that its values are always less than or equal to those of DSC but Tables 2 and 4 show that WDC has very often higher values than the other two coefficients. The order of these masks would be nearly the same, regardless of whether they were ranked by their DSC, WDC, or LDC values. While there were some differences in the order of the mean scores of the two-dimensional masks forming these three-dimensional masks and three Dice coefficients, it should be noted that this difference might be because of different numbers of truly positive pixels in the slices of the three-dimensional masks. Spearman’s rank correlation coefficient was 0.899 (*p*-value: 8.96e$-$7) between the DSCs of the predicted three-dimensional segmentation mask and the related mean scores computed from the transaxial masks, 0.941 (*p*-value: 1.83e$-$8) between the WDCs and the mean scores, and 0.911 (*p*-value: 3.51e$-$7) between the LDCs and the mean scores.

The two human evaluators gave the same score for 163 cases out of 190, resulting in an inter-rater agreement of 85.3%. For all the segmentation masks, the numerical difference between the scores given by the first and the second evaluator was at most 1, and the average difference was 0.147. The Spearman’s correlation coefficient in the scores between the evaluators was 0.956 (*p*-value: 2.11e$-$102). The relative differences between DSC, WDC, and LDC stayed the same when the scores given by the second evaluator were considered ground truth. For instance, Spearman’s correlation coefficient was 0.843 between the DSCs and the scores by the second evaluator, 0.902 for WDCs, and 0.875 for LDCs.

## Discussion

In this article, we introduced two different modifications of the DSC for tumor segmentation from PET images. In earlier research, a few different modifications of the Dice coefficient have been introduced: For instance, Musial et al. [19] evaluated segmentation of laser ophthalmoscope capillary perfusion images by considering all the FP pixels less five pixels away from the ground-truth positive pixels as TP pixels, Morra et al. [20] penalized matching based on the ratio of the sizes of the predicted and the correct segments, and Hecksel et al. [21] used a DSC extended for measuring the similarity between more than two segmentation masks. However, to the best of our knowledge, the two modifications introduced here, WDC and LDC, are fully novel.

Our tests suggest that WDC in particular might be of use for evaluating predicted masks for medical images. Compared to the usual DSC, this weighted coefficient produced significantly less variation for such segmentation results that were visually evaluated to be good or excellent. It also produced a higher correlation with the scores given in the visual evaluation to both two-dimensional and three-dimensional predicted masks than the other two coefficients. In our experiment, the WDC values higher than 0.85 meant that there were neither fully FP or FN components, or in other words, that the CNN detected all the cancerous regions and did not incorrectly classify healthy targets, such as parts of the brain, as positive. This threshold value naturally depends on the choice of the constant and the environments used when computing WDC, though.

However, it should be noted that WDC sometimes gives very small positive values for segmentation masks that were visually evaluated to be fully wrong. Given the definition of WDC, this is because the components predicted as positive are close to the real tumors while their location is wrong. Additionally, it should be noted that WDC is not perhaps well-suited for evaluating which of two segmentation predictions is better than the other if they both have very small WDC values. Namely, if we change the classification of a correctly classified negative point as positive in a segmentation mask with a WDC value small enough, and this point is close enough to the real segment to be included in its weighted environment, then the WDC value increases. However, this issue could be solved by some hybrid method of setting the values of WDC to 0 if the original DSC is 0.

In our experiments, the numerical values of LDC were quite close to those of DSC. To develop this type of coefficient that uses the elements of the false components as a penalty, it might be necessary to choose the environment differently. Furthermore, a coefficient greater than 1 could be added for the loss term in the denominator so that this loss term would have more significant weight to the final value of the quotient. One possible idea for further study would be to create another coefficient that uses both the weighted environments of WDC and the loss term of LDC.

Additionally, the choice of the numerical parameters related to the weights of WDC should be considered carefully. Namely, the WDC values are not directly comparable unless all the parameters are the same, but the image resolution and the potential cancer type should be taken into account while selecting these parameters. Namely, it affects the values of our DSC modifications how many pixels there are in the images between the correct and the predicted segments, and how large the segments are in pixels. Establishing standardized values for these numerical parameters is naturally important, but more research about this topic with additional datasets on different cancer types would be required in order to suggest some default parameter choices. Our current choices were chosen due to the fact that we believed that the segments annotated by two different doctors could easily differ by a few voxels, even if both doctors had experience and knowledge on how to annotate tumors correctly. Further study is warranted on the sensitivity of numerical parameter choices for WDC and LDC, and also their generalizability across different datasets and cancer types.

## Conclusion

We introduced two different modifications of the Dice score, in order to find a coefficient whose values would correspond better with the quality of the segmentation masks evaluated visually. Out of these modifications, one was based on assigning different weights to the elements surrounding the original segments and the other used an additional loss term as a penalty for false segmentation too far away from the correct segments. According to our results, our weighted coefficient might be a clinically more meaningful metric than the traditional Dice coefficient, especially in oncology research or in any studies based on PET imaging.

## Data Availability

Patient data is not available due to ethical restrictions but code is available at github.com/rklen/Modified_Dice_coefficients
